# A Novel Hydrogel Sponge for Three-Dimensional Cell Culture

**DOI:** 10.3390/pharmaceutics16101341

**Published:** 2024-10-19

**Authors:** Sara Baldassari, Mengying Yan, Giorgia Ailuno, Guendalina Zuccari, Anna Maria Bassi, Stefania Vernazza, Sara Tirendi, Sara Ferrando, Antonio Comite, Giuliana Drava, Gabriele Caviglioli

**Affiliations:** 1 Department of Pharmacy (DIFAR), University of Genoa, 16148 Genoa, Italy; sara.baldassari@unige.it (S.B.); giorgia.ailuno@unige.it (G.A.); guendalina.zuccari@unige.it (G.Z.); giuliana.drava@unige.it (G.D.); 2 The Brain Cognition and Brain Disease Institute, Shenzhen Institute of Advanced Technology, Chinese Academy of Sciences, Shenzhen 518000, China; my.yan@siat.ac.cn; 3 Department of Experimental Medicine (DIMES), University of Genoa, 16132 Genoa, Italy; anna.maria.bassi@unige.it (A.M.B.); stefania.vernazza@unige.it (S.V.); tirendisara@gmail.com (S.T.); 4 Inter-University Center for the Promotion of the 3Rs Principles in Teaching & Research (Centro 3R), 56122 Pisa, Italy; 5 Laboratory of Comparative Anatomy, Department of Earth, Environmental, and Life Sciences (DISTAV), University of Genoa, 16132 Genoa, Italy; sara.ferrando@unige.it; 6 Department of Chemistry and Industrial Chemistry (DCCI), University of Genoa, 16146 Genoa, Italy; antonio.comite@unige.it; 7 IRCCS Ospedale Policlinico San Martino, 16132 Genoa, Italy

**Keywords:** scaffold, 3D cell culture, polyacrylic, hydrogel sponge, cancer cell culture, drug testing, thermal treatment

## Abstract

Background/Objectives: Three-dimensional (3D) cell culture technologies allow us to overcome the constraints of two-dimensional methods in different fields like biochemistry and cell biology and in pharmaceutical in vitro tests. In this study, a novel 3D hydrogel sponge scaffold, composed of a crosslinked polyacrylic acid forming a porous matrix, has been developed and characterized. Methods: The scaffold was obtained via an innovative procedure involving thermal treatment followed by a salt-leaching step on a matrix-containing polymer along with a gas-forming agent. Based on experimental design for mixtures, a series of formulations were prepared to study the effect of the three components (polyacrylic acid, NaHCO_3_ and NaCl) on the scaffold mechanical properties, density, swelling behavior and morphological changes. Physical appearance, surface morphology, porosity, molecular diffusion, transparency, biocompatibility and cytocompatibility were also evaluated. Results: The hydrogel scaffolds obtained show high porosity and good optical transparency and mechanical resistance. The scaffolds were successfully employed to culture several cell lines for more than 20 days. Conclusions: The developed scaffolds could be an important tool, as such or with a specific coating, to obtain a more predictive cellular response to evaluate drugs in preclinical studies or for testing chemical compounds, biocides and cosmetics, thus reducing animal testing.

## 1. Introduction

Efficacy and toxicity studies are required in many chemical technological fields for pure substances and formulations. Health and environmental surveillance and regulatory agencies around the world are urging for the development of alternative methods to replace in vivo tests on animals in the aforementioned areas. Thus, more reliable technologies for cell culture to mimic the in vivo biological environment and crosstalk among different cells/tissues/organs need to be developed and applied worldwide.

Three-dimensional (3D) cell culturing was devised to overcome two-dimensional (2D) culture response defects, as this allows cells to grow in vitro in all directions, closely resembling in vivo conditions [1,2]. In actual fact, 3D cell culture systems allow the investigation and understanding of complex cellular physiological mechanisms and are therefore useful in pharmaceutical assays, differentiation studies, cancer biology research and tumor models, and gene and protein expression studies, as well as being applied in tissue engineering, bioreactors and the in vitro modelling of human organs [3,4]. Moreover, 3D cell cultures exhibit higher stability and longer lifespan than 2D cell cultures; therefore, they might be more appropriate for toxicology studies and for demonstrating the long-term effects of drugs.

The commercially available culturing tools for 3D cell culture can be classified in two types: scaffold techniques and scaffold-free techniques [5]. Scaffold-free techniques employ approaches independent of the use of scaffolds, such as hanging drops/gravity culture plates, rotation culturing (e.g., spinner flasks) and spontaneously formed 3D cultures. Conversely, scaffold-based techniques involve condensed supports that may be classified as solid structures and hydrogel matrixes.

Hydrogel scaffolds (HSs) are made of hydrophilic polymer networks that can easily mimic extracellular matrix, therefore being more favorable for organotypic cell proliferation [6].

The incorporation of specific agents to tailor their properties is made possible by their versatility and the ability to modify their composition using current technologies. The tunability of hydrogels allows them to be customized for various fields, including agriculture, biomedicine and environmental science [7,8,9,10]. Meanwhile, their major drawback is inadequate mechanic strength, having too weak a consistency, or, in the case of stiffer hydrogels, they may be too viscous, hindering cell migration; overall, they are limited in scale-up because of poor long-term storage issues and stability.

Instead, solid HSs can be manufactured in a controlled and reproducible fashion, can be appropriately molded, are inert in structure and have long term stability. Another important advantage is that cells can freely migrate throughout the structure without much resistance. In addition, the mechanical stability of solid porous scaffolds makes them easy to handle, which is more difficult with soft hydrogels. Therefore, the mechanical stability of these HSs, along with their high porosity and interconnectivity, make them ideal for 3D cell cultures. Nevertheless, solid scaffolds present some drawbacks, like complex cell recovery and poor light transmission, which limits cell observation during culture growth.

Three-dimensional HSs can be prepared by different techniques including salt leaching [11], phase separation, freeze drying, gas foaming, emulsion templating and 3D printing [12]. Phase separation is a method allowing the production of biocompatible scaffold matrices by the precipitation of polymers from a polymer-poor and a polymer-rich phase. This method has the advantage of involving a very simple process and of requiring minimal apparatus [13]. Freeze-drying has been widely used to prepare porous scaffolds for tissue engineering and other biological applications. The low surface tension involved during the freeze-drying process leads to the maintenance of the pore structure (particularly mesopores and micropores); moreover, this technique allows to avoid the use of toxic organic solvents, while the low temperature of the process helps to maintain the activity of biomacromolecules [14]. Gas foaming avoids the use of solvents, and is therefore particularly appropriate for incorporating sensitive molecules into matrices without drastically reducing their bioactivity [15]. Emulsion templating is a convenient route for the fabrication of matrices with up to 99% porosity and high interconnectivity [16], besides exhibiting advantages like the ease of control of porosity by controlling emulsion droplet size, relatively rapid structure formation in bulk, and cost-effectiveness. Finally, 3D printing allows the production of scaffolds by stacking materials layer by layer, obtaining structures with customized shapes and porosities [17]. From a manufacturing point of view, preparing porous structures through polymer melt or by sintering compacted powders are more convenient routes, as they allow the rapid and economic production of HSs of different shapes and sizes [18].

Many polymer types have been tested for 3D scaffold preparation, such as polystyrene, collagen, alginate, agarose, etc. The main issue is the compatibility of the polymer with the cultured cells.

Here, we describe the preparation and characterization of a transparent sponge-like HS obtained by a new procedure based on thermal treatment associated with template salt-leaching technique [19,20]. The properties of this HS were explored by response surface methodology based on experimental design for mixtures. In addition, biocompatibility and suitability for cell culturing of different cell lines were assessed and reported.

## 2. Materials and Methods

### 2.1. Materials

Carbopol 980 NF (CBP980), Carbopol 974P NF (CBP974) and Noveon^®^ Polycarbophil (POL) were obtained from Lubrizol (Wickliffe, OH, USA); sodium hydrogen carbonate, sodium chloride and all other reagents used for the experiments were of analytical grade and purchased from Merck (Darmstadt, Germany).

### 2.2. Preparation of 3D Scaffolds

A suitable amount (approx. 2 g) of sodium chloride (NaCl) was milled in a 1.5 mL stainless steel grinding jar containing 3 stainless steel 5 mm diameter balls using a high oscillating frequency mill (Mixer Mill MM2, Retsch, Haan, Germany) at a vibrational frequency of 8 Hz for 30 min. NaCl and CBP980 (or CBP974 or POL) powders were sieved between 250 and 125 µm in ASTM certified test sieves (Giuliani, Torino, Italy) on an analytical sieve shaker (AS200 Retsch, Haan, Germany), while sodium hydrogen carbonate (NaHCO_3_), with particle sizes of under 250 µm, was used as received. The homogeneous mixing of the three components was conducted in a stainless steel jar of suitable volume, using a Turbula mixer (WAB, Basel, Switzerland). Approx. 300 mg of the mixture was compressed in a 13 mm diameter evacuable stainless steel die, using a hydraulic press (PerkinElmer, Waltham, MA, USA) applying a force of 8800 N for 15 s. The obtained tablets were thermally treated in a forced-air oven (GC HP 5890 series II, Hewlett-Packard, Palo Alto, Santa Clara, CA, USA), employing the following temperature program: heating from 30 °C to 150 °C at 30 °C/min, undertaking a isotherm step for 15 min, then cooling to room temperature by forced-air ventilation. After the thermal treatment, the size of each compacted unit was measured and the percentage of loss of weight (W_t_%) was calculated as:

W_t_% = (w_0_ − w_t_)/w_0_ × 100 (1)

where w_0_ is the initial weight and w_t_ is the weight after thermal treatment.

In the leaching step, the thermally treated compacts were swollen in 800 mL of deionized water under magnetic stirring, replacing the water several times until the compacted units reached their maximum size and no residual salts could be detected in the wastewater. The presence of sodium ions was measured potentiometrically (Compact Titrator G20 Mettler Toledo, Greifensee, Switzerland) using an ion selective electrode (perfectION comb Na^+^) on 20 mL of washing water, while the absence of chloride ions was assayed by the silver nitrate test. The swollen scaffolds, conditioned in a suitable buffer, were cut at different shapes and sizes.

Table 1 shows the HSs prepared according to the experimental design described in Section 2.9.

### 2.3. HS Lyophilization Procedure

The lyophilization procedure for characterization studies is described in the Supplementary Materials.

### 2.4. HS PBS Conditioning and Sterilization

After lyophilization, the scaffolds were conditioned in PBS, a buffer commonly used as component of cell culture medium, up to constant dimensions. The conditioned HSs were sterilized in borosilicate glass vials sealed by an aluminum crimped cap using a saturated steam autoclave (Sterilplus Vacuum De Lama, Pavia, Italy), then heated for 30 min under a saturated steam pressure of 98 kPa over atmospheric pressure, at 120 °C.

### 2.5. Swelling Study on Pure Polyacrylic Polymer Tablets

Pure polymeric matrices, submitted to thermal treatment at 150 °C for 15 min as previously described, were placed in 700 mL of water (or other media used) and kept under stirring conditions (50 rpm) at 37 °C. At established time periods, the tablets were withdrawn, and their dimensions and weights were recorded. The swelling index (SI) of the matrices was measured as follows:

SI = (w*_i_* – w_t_)/w_t_ × 100 (2)

where w*_i_* is the weight of the swollen scaffold at time *i* and w_t_ is the tablet weight after thermal treatment.

### 2.6. Field Emission Scanning Electron Microscopy (FESEM)

A FESEM (field emission scanning electron microscope—ZEISS Supra 40 VP, Carl Zeiss STM, Oberkochen, Germany) was employed for microstructural characterization, acquiring the micrograph cross-sections of lyophilized HSs. For measurement, the samples, placed on a stub using double-sided conducting tape, were coated with a thin Au layer (about 15–20 nm) by sputtering.

### 2.7. Porosity Study by Physisorption of N_2_ at 77K

A sample of water-conditioned lyophilized HS was submitted to adsorption/desorption measurements (under N_2_ at 77 K) using an ASAP 20l0 porosimeter (Micromeritics, Norcross, GA, USA). Pore size distribution was obtained by the BJH method implemented in the instrument proprietary software (ASAP2010 V4.00).

### 2.8. Transparency and Optical Properties

A PBS-conditioned HS was immersed in PBS in a Petri dish placed over printed paper. The readability of the printed graphic signs through the scaffold and through neat PBS were compared.

The UV–VIS transmittance of the scaffolds was measured on a water-conditioned scaffold in comparison with non-porous hydrogels obtained by thermally treated pure polymer compacts swollen in water.

### 2.9. Experimental Design and Response Surface Methodology

An experimental design for mixtures [21] was applied, and for each formulation, the following responses were measured: Young’s modulus (kPa), YM; yield strength (kPa), YS; apparent density (mg/cm^3^), ρapp; compression density (mg/cm^3^), ρcomp; swelling index (%), SI; porosity by density method, εdm; porosity by liquid displacement method, εld; carboxylate (% on the total number of carboxylic groups), COO^−^; weight loss after thermal treatment (%), W_t_%; diameter variation after thermal treatment (%), Δdt; and thickness variation after thermal treatment (%), Δht. More details are provided in the Supplementary Materials.

### 2.10. Mechanical Properties 

The mechanical properties of the hydrated HSs were evaluated by compression tests on an LRX dynamometer (Lloyd Instruments, Bognor Regis, UK) at 25 ± 2 °C. The following values were measured on six HSs for each scaffold composition:

- YM = Young’s modulus (kPa) measured as the slope of the linear part of the stress–strain curve preceding the plastic deformation at YS.
- YS = yield strength (kPa), measured as the stress causing the minimum irreversible deformation in the material.

More details are provided in the Supplementary Materials.

### 2.11. Apparent and Compression Density Study

The measurement of apparent and of compression density are described in the Supplementary Materials.

### 2.12. HS Carboxylic Acid Group Titration

An accurately weighed amount of milled lyophilized HS was kept in 25.0 mL of 0.1 N NaOH aqueous solution overnight to salify all the carboxylic groups. Then, the HS was removed and the solution was titrated with 0.1 N HCl aqueous solution. The same volume of neat 0.1 N NaOH solution was titrated with the same HCl solution: the COOH milliequivalents present in the HS were obtained from the difference in consumed HCl milliequivalents.

The carboxylate ions (meq) present were obtained following the same procedure: in this case, the lyophilized HS, conditioned with 25.0 mL of 0.1 N HCl solution, were titrated with 0.1 N NaOH. The results are reported as milliequivalents of carboxylic acid or carboxylate ion, referred to the weight (mg) of lyophilized scaffold (meq/mg). The percentages were calculated as follows:

COOH (%) = meq/mg COOH/(meq/mg COOH + meq/mg COO^−^) × 100 (3)

COO^−^ (%) = meq/mg COO^−^/(meq/mg COOH + meq/mg COO^−^) × 100 (4)

### 2.13. HS Porosity

To determine the porosity, two methods were followed. Before the porosity measurement, the scaffolds were conditioned and sterilized in PBS, followed by rewashing with water until the complete elimination of sodium ions was achieved, and then lyophilized.

The first method is based on density measurement [22]. Using a caliper, the radius and the height of a lyophilized HS of cylindrical shape were measured, followed by the calculation of the volume and the recording of the apparent density (ρ_1_). After cutting the HS into smaller pieces, a weighted amount of it was put into a die of 13 mm diameter and, using a hydraulic press under vacuum, compacted applying a compression force of 8 tons for 15 min. Finally, after the measurement of the volume and weight of the obtained tablet and the estimation of its density (ρ_2_), the scaffold porosity (εdm) was calculated using the following equation:

εdm = 1 − ρ_1_/ρ_2_ × 100 (5)

A modified liquid displacement method was also used to determine the porosity of scaffolds [23,24,25]. The HSs used for porosity measurement were all conditioned and sterilized in PBS, washed with water and lyophilized. Acetonitrile was used as the displacement medium because it permeates through the matrix without swelling or shrinking it.

The following equation was applied to calculate HS porosity (εld):

εld = V_p_/(V_p_ + V_s_) × 100 (6)

where V_p_ is the pore volume calculated as follows:

V_p_ = (W_w_ − W_d_)/δ_acetonitrile_ (7)

where W_w_ and W_d_ were the weights of wet and dried scaffolds, respectively, and V_s_ is the volume of a 13 mm diameter tablet of cylindrical shape, obtained through compression of the dried HS in a 13 mm diameter cylindrical die by applying the force of 8 tons for 15 min with a hydraulic press; a vacuum pump connected to the die allowed the elimination of the air. All experiments were carried out in triplicate. More details are given in the Supplementary Materials.

### 2.14. Molecular Diffusion 

The molecular diffusion through the HS at 37 °C was evaluated in a horizontal Franz diffusion cell apparatus, using metformin hydrochloride (MH) as a small, very hydrophilic and soluble diffusing test molecule. 

The diffusion coefficient D was calculated by the following equation [26]:

(8) $$D=\frac{1}{\beta \;t}\ln\left(\frac{C_{1}(t)-C_{2}(t)}{C_{1}^{0}-C_{2}^{0}}\right)$$

where:

(9) $$\beta =\frac{A_{H}}{W_{H}}\left(\frac{1}{V_{1}}+\frac{1}{V_{2}}\right)$$

$C_{1}(t)$ = concentration of MH in donor compartment after time *t*; $C_{2}(t)$ = concentration of MH in receptor compartment after time *t*; $C_{1}^{0}$ = initial concentration of MH in donor compartment; $C_{2}^{0}$ = initial concentration of MH in receptor compartment; A_H_ = effective cross-sectional area of diffusion in the HS; W_H_ = thickness of the HS; $V_{1}$ = volume of donor compartment; $V_{2}$ = volume of receptor compartment.

By plotting $\ln\left(\frac{C_{1}(t)-C_{2}(t)}{C_{1}^{0}-C_{2}^{0}}\right)$ gainst time *t*, the ordinary least square regression line was obtained; the slope was used to calculate the diffusion coefficient D.

More details are provided in the Supplementary Materials.

### 2.15. Cell Culture Protocol

Two human cell lines, HeLa from cervix epidermoid carcinoma, as model of undifferentiated cells, and HECV normal endotheliocytes from the umbilical cord (certified by STR DNA profile analysis by Biological Bank, a Core Facility of the IRCCS San Martino University Hospital—IST National Institute for Cancer Research (Genoa, Italy)), were routinely cultured at 37 °C under 5% CO_2_ in Dulbecco’s Modified Eagle’s Medium (DMEM) plus 10% heat-inactivated fetal bovine serum.

Neither antibiotic nor antifungal solutions were added to standard or experimental medium to avoid any potential interference of these drugs with the cell culture conditions. All cell cultures were found to be mycoplasma-free on regular checks with the Reagent Mycoplasma Set (Euroclone).

The population doubling time for HeLa and HECV was estimated to be 16 and 17 h, respectively, by using a user friendly software (Roth V. 2006 Doubling Time Computing—available at www.doubling-time.com/compute.php).

The HSs were placed into each well of a 24-well plate, then PBS was replaced with standard culture medium overnight. HeLa and HECV cells were seeded at 2 × 10^5^ cells/well/20 µL standard medium. After 24 h, a further 200 µL of standard medium were added in each well. The cell culture medium was changed every 2–3 days by removing it from the bottom of the wells by gentle aspiration using a vacuum manifold.

### 2.16. Cell Viability Test

For in vitro biocompatibility and viability studies, 9 mm diameter cylindric HSs, conditioned and sterilized in PBS, were used.

As a first step, it was investigated whether the HSs were suitable to sustain the most common viability test assay performance, by putting HSs, without cells, into 6 multi-well plates and adding MTT, MTS or NRU viability dye. After a 3 h incubation time at 37 °C, all the dyes spread into gels without a change in color, confirming no interference with the viability dyes. The health states of HeLa and HECV cells, in terms of their metabolic activity, were measured by the Alamar Blue (Invitrogen, Thermo Fisher Scientific Inc., Waltham, MA, USA) assay over 20 days, performed every 4 days on each culture.

The cells inside the scaffolds were observed using a phase-contrast microscope (Leica DMIRB HC Fluo 257041; Leica Microsystems Srl S.p.A., Milano, Italy).

More details are given in the Supplementary Materials.

### 2.17. Test with Positive Irritation Compound

To assess the suitability of HSs as a scaffold for an in vitro model to predict the irritative potential of a chemical compound, HeLa cells, cultured for 10 days in C7 and C8 HSs, were submitted to an MTS viability test after 3 h of exposure to 0.5 and 5 mM aqueous solutions of NiSO_4_ as the positive irritation agent.

The viability index was assessed by MTS assay; cell viability was expressed as the percentage of vital cells versus the respective untreated culture.

### 2.18. Fluorescent Imaging of Cells in Agarose-Embedded HS

Cell suspensions were embedded in HSs at two different densities for well (1 and 2 × 10^5^ cells).

After fixation in paraformaldehyde (PAF) (4% in PBS, 8‰ NaCl and pH 7.4) for 24 h at 4 °C, the HSs were gently washed 3 times with PBS at room temperature under gentle agitation. The HSs were then embedded in agarose 0.8% in PBS (0.8 g of agarose powder was dissolved through gentle agitation in 100 mL of PBS, heated to boiling, then cooled to 55/56 °C and used immediately). The embedded HSs were maintained at 4 °C in the dark and were hydrated with PBS until the cutting operation, which was performed using an appropriate vibratome with a thickness of 40 μm in a PBS bath; the sections were immediately mounted on histological slides.

To visualize cell distribution and nuclear shape, and to test the suitability of the HS with fluorescence microscopy, two commonly used nuclear acid fluorescent stains, propidium iodide (PI) and 4′,6-diamidino-2-phenylin-dole (DAPI), were used.

More details are provided in the Supplementary Materials.

### 2.19. Statistical Analysis

NEMRODW software (LPRAI, Marseille, France) was used for experimental design and for the related statistical analysis and graphs. Data were analyzed by principal component analysis, two-sample t-tests or one-way ANOVA and post hoc tests, using Systat for Windows ver. 13 (Systat Software Inc., San Jose, CA, USA). The confidence level was set at *p* = 0.05 if not otherwise indicated.

## 3. Results and Discussion

The rationale for the development of this scaffold for the 3D cell culture was based on certain property changes observed in polycarbophil (POL), a poly(acrylic acid) polymer crosslinked with divinyl glycol [27]. This polymer, when subjected to thermal treatment, showed a change in the morphology and properties of the powder granules in a narrow and well-defined temperature range. This phenomenon has been previously exploited to produce a matrix capable of controlling the drug release from a tablet [27]. In fact, POL generates a matrix when the powder is compacted in a tablet and submitted to thermal treatment. We observed the same behavior in other acrylic acid polymers crosslinked with different agents like allyl sucrose or allyl pentaerythritol, known with their proprietary name of Carbopol™. Compacts of Carbopol 980 (CBP980 coded as C) or Carbopol 974P (CBP974, coded as K) submitted to the same thermal treatment of POL (coded as P) formed a monolithic matrix, gelling after hydration in aqueous medium. It is probable that at the base of this phenomenon lies the same mechanism elucidated for POL [27]. All the three polyacrylate polymers used in this study are cross-linked: Carbopol with allyl pentaerythritol and Polycarbophil with divinylglycol. Carbopols share the same values of pKa (6.0 ± 0.5), carboxylic acid content (56–68%) and glass transition temperature (105 °C) but differ in their viscosity; indeed, the viscosity of Carbopol 974 0.5% *w*/*w* is 29,400–39,400 cP and the one of Carbopol 980 0.5% is 40,000–60,000 cP [28]; therefore, the mean chain length of the latter is supposed to be higher. However, the exact molecular weight cannot be measured, and the relevant brochures report a value close to up to 4.5 billion for both, due to the multiple interlinkage of the cross-linkers [29].

**Figure 1:**
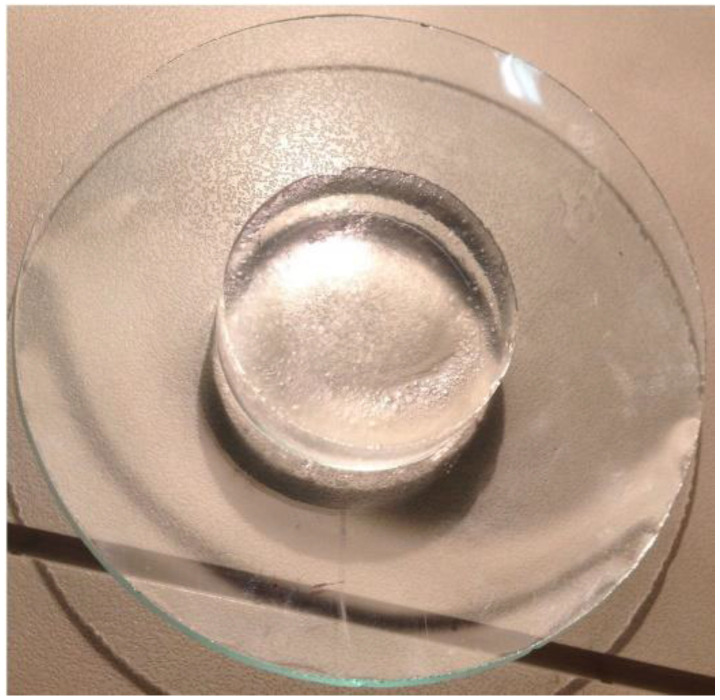
Pure CBP980 cylindrical compact (300 mg; 13 mm diameter) thermally treated at 150 °C for 15 min: after 23 h swelling study in phosphate buffer pH 7.2 at 37 °C, it reached 5 cm diameter.

**Figure 2:**
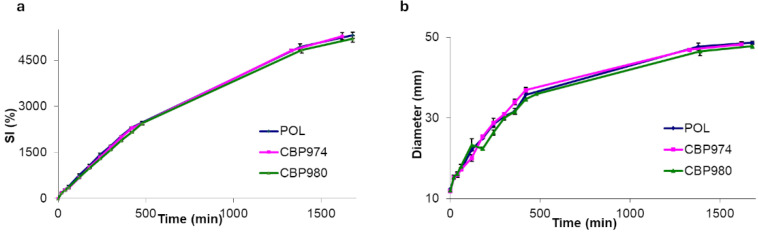
SI (**a**) and diameter (**b**) change profiles of 12 mm diameter and 300 mg compacts of pure polyacrylic acids submitted to thermal treatment at 150 °C for 15 min during swelling in phosphate buffer at 37 °C.

In fact, tablets of the pure polymers were treated at 150 °C for 15 min and put in aqueous medium where they swelled completely, producing a transparent cylindrical hydrogel matrix with a 4-fold increase in diameter and a SI higher than 5000% with the absorption of more than 15.5 g of water (Figure 1 and Figure 2). Differently, the same untreated compacts did not form an unerodible matrix and disaggregated during the swelling study.

The consideration that hydrogels with properties similar to the extracellular matrix are used in cell culture [30], and that the unerodible gels obtained by the procedure above described have good optical and mechanical properties, inspired the idea of exploiting these matrices for producing scaffolds for 3D cell culture.

As the gels obtained from pure polymers are highly viscous (at 25 °C CBP980 0.5% 53,400 mPa·s; CBP974 0.5% 29,750 mPa·s; POL 0.2% 9940 mPa·s, according to the manufacturer’s relevant certificates of analysis), making them not suitable for the growth of a 3D cell culture, we conceived the idea of creating pores within the matrix, applying the template technique based on the combined use of a thermally induced gas-forming agent and thermally forming the matrix polymer above described using the salt-leaching technique. For their easy availability and low cost, NaCl and NaHCO_3_ were chosen as the salt-leaching and gas-forming agents, respectively. NaHCO_3_ was selected because at the temperature of the treatment (around 150 °C), it undergoes a decomposition to Na_2_CO_3_(s), H_2_O(g) and CO_2_(g) [31].

This manufacturing procedure, if compared to phase precipitation, allows us to achieve better yields; it generates a much more interconnected scaffold in comparison to gas-foaming processes and can more easily be conducted in comparison to emulsion templating, which is frequently affected by emulsion instability; finally, salt-leaching is generally cheaper when compared to 3D printing, which also frequently involves complex post-processing cleaning to remove impurities noxious to cells.

**Figure 3:**
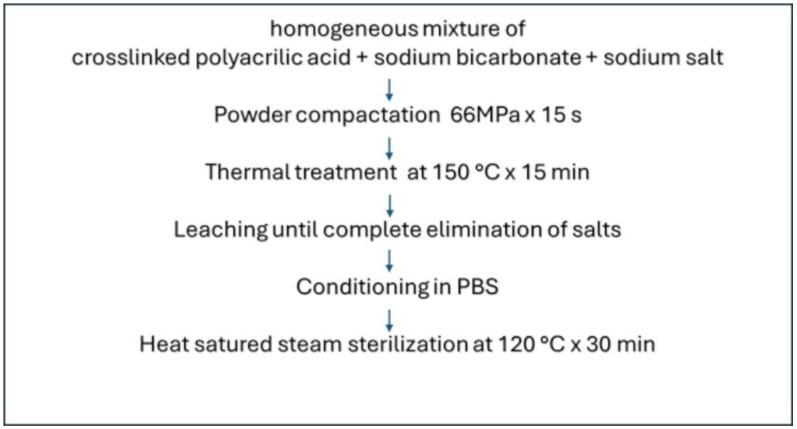
Manufacturing steps for HS preparation.

The different steps of HS manufacturing are reported in Figure 3.

To uniformly distribute the components in the matrix and to obtain a suitable final scaffold pore size, the powder components were used in dimensional range from 125 to 250 μm. The compacted powder was submitted to the isothermal treatment conditions, at 150 °C for 15 min, which resulted as adequate for matrix formation and CO_2_ evolving.

**Figure 4:**
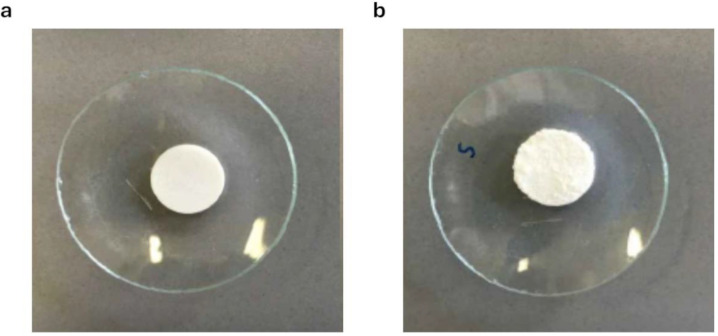
Formulation C0 before (**a**) and after (**b**) thermal treatment (150 °C for 15 min).

In Table 2, the morphological characteristics of three compact units prepared with the same proportions (40% of polymer, 30% of NaHCO_3_ and 30% of NaCl), but using the three different polymers, before and after thermal treatment are compared. The thermal treatment determined a size increase in the compacted units, associated with weight loss, which is especially evident (*p* < 0.05) when using CBP980 (formulation coded as C), while the surface of the compacts became rough because of CO_2_ evolving (Figure 4). The density of the compacted units decreased (by approx. 70% for C0) because of the thermal treatment.

The treated compacts were then submitted to the salt-leaching phase in water. The final point of leaching was determined after complete matrix swelling, when the hydrogel matrix reached constant size and the residual sodium and chloride ions were no longer detectable in the washing/leaching water. 

The leaching determines an increase in the diameter and thickness for hydrogel matrix formation during the swelling of thermally treated units. The diameter and thickness of C0, for example, increased several times with respect to the original compact dimensions (Table 2). 

**Figure 5:**
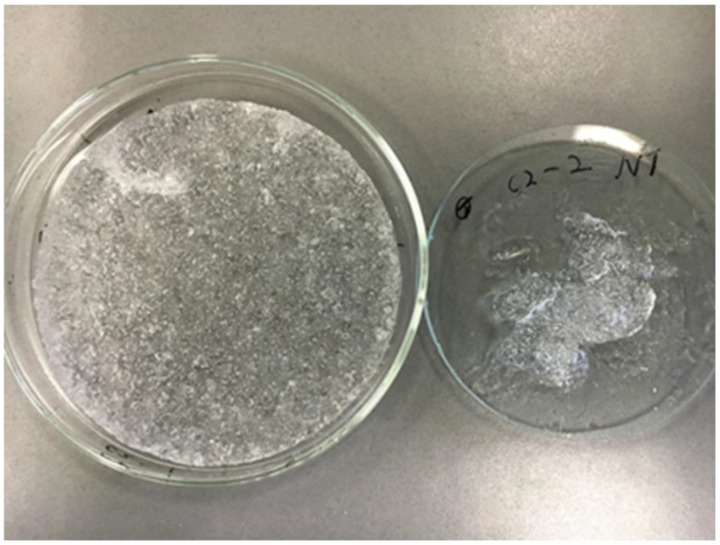
Comparison between a C0 hydrogel submitted to thermal treatment (on the left) and an untreated C0 (on the right) after the leaching step.

As evident in Figure 5, the compacted units not submitted to the thermal treatment disintegrated during the leaching step. 

**Figure 6:**
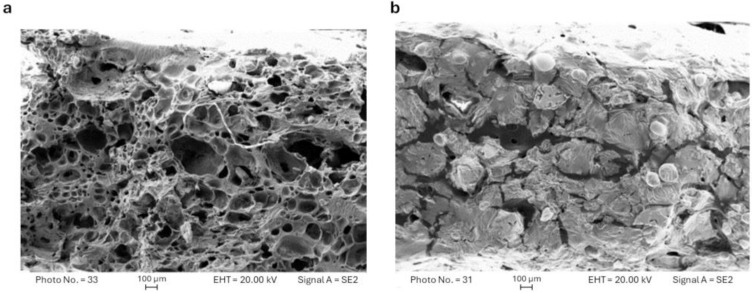
FESEM micrograph of cross section of a thermally treated C0 compacted unit (**a**) and of an untreated compacted unit (**b**).

In FESEM micrographs, the generation of a continuous and porous structure is evident in a thermally treated compacted unit (Figure 6a) in comparison to an untreated one (Figure 6b), which explains the unerodable hydrogel matrix formation when the compact undergoes leaching.

**Figure 7:**
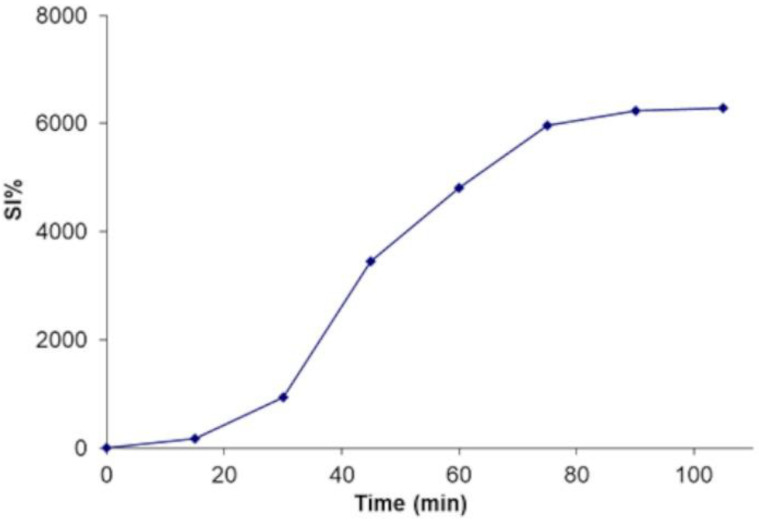
Swelling profile of C0 thermally treated compact unit during leaching in water.

**Figure 8:**
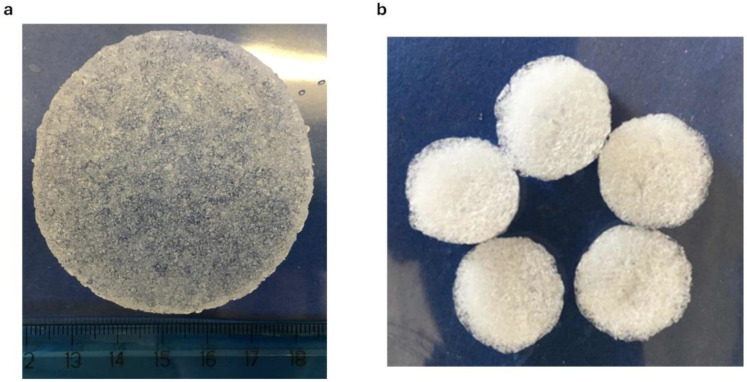
C0 hydrogel scaffold after leaching phase (**a**) and after lyophilization (**b**).

During the approx. 70 min leaching step, the scaffold swelled, reaching a SI of about 5000% (Figure 7), assuming the structure and consistence of a hydrogel sponge (Figure 8a). The soft spongy structure becomes visible in the scaffold after lyophilization, when it appears as a highly trabecular dry white sponge (Figure 8b). When placed in aqueous medium, the lyophilized scaffold swells up to reconstitute the originating unerodible hydrogel matrix, retaining the morphological and mechanical properties suitable for culturing cells.

**Figure 9:**
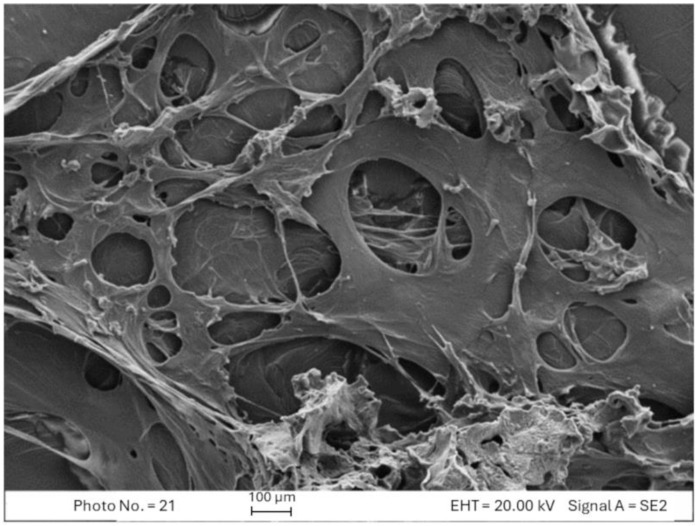
FESEM micrograph of cross-section of lyophilized C0 matrix corresponding to the same scaffold reported in Figure 8.

After lyophilization, the distributions and size of the pores, ranging from 100 to 200 µm, are similar to the ones of the swollen scaffold ready for culturing the cells (Figure 9).

In this matrix, the swelling is related to the polycarboxylic nature of the polymeric component. In fact, the electrostatic repulsion between carboxylate groups on the polymer backbone is the driving force of the swelling process. The swelling extension is related to the pH of aqueous medium in which the scaffold is hydrated or conditioned. The micrograph of a lyophilized scaffold in Figure 9 clearly shows the mechanical tension for the swelling of the hydrogel in water.

**Figure 10:**
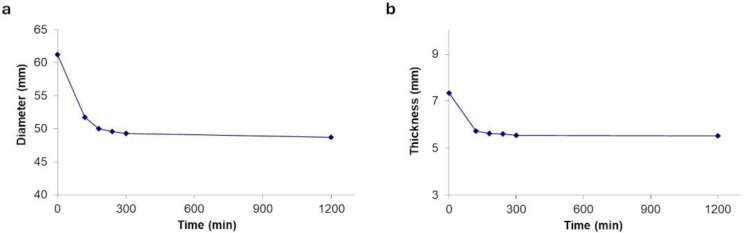
Diameter (**a**) and thickness (**b**) variation during PBS conditioning for C0 scaffold during leaching in water.

For this reason, the scaffolds, before being used for biological studies, must be conditioned with PBS or another appropriate medium. As shown in Figure 10, the diameter and thickness of C0 scaffold progressively decreased when the matrix was placed in PBS, with the minimum reached after 3 h, corresponding to the time necessary to saturate the matrix with PBS.

Another important feature of this scaffold for cell culture is its sterilizability. Rarely polymeric scaffolds can be sterilized by thermal processes, which can damage the polymeric skeleton, as well as other energetic processes such as ionizing radiation or chemical sterilization; moreover, chemical sterilization could contaminate the hydrogel with toxic residuals incompatible with cellular culture. For this reason, the large majority of 3D scaffolds require expensive aseptic production procedures [32].

**Figure 11:**
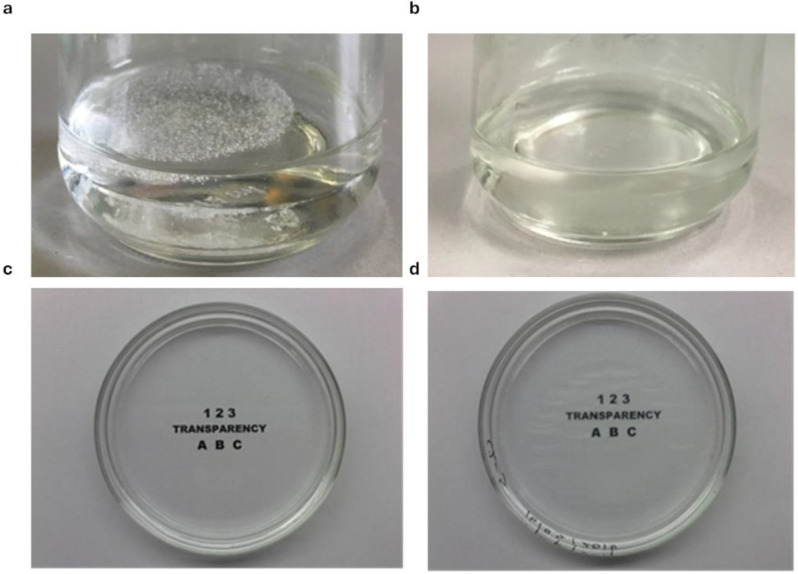
C0 scaffold before (**a**) and after (**b**) sterilization; dish filled with neat PBS placed on the script test as blank (**c**); dish filled with HS and PBS placed on a script test (**d**).

Sterilizability was verified by placing the scaffolds, suitably conditioned with PBS, in sealed pressure-resistant closed vials containing the buffer and submitting them to a moist heat sterilization procedure. Sterilization did not cause any variation of the scaffold weight or size, with respect to the PBS-conditioned scaffolds not undergoing the sterilization procedure (*p* > 0.05). After sterilization, the scaffolds could be stored in the sterilization vial for at least 12 months, without changes in their features or presence of microbial contamination. After moist heat sterilization, the scaffold became perfectly transparent, as shown in Figure 11a,b, for the evacuation of the carbon dioxide that had remained included in the newly formed matrix. It is likely that the release of gas, at the sterilization pressure, generated the breaking of some septa between pores, improving the scaffold interconnectivity. Thus, the sterilization step also plays a functional role in the process of scaffold production. The high optical transparency of these scaffolds is noteworthy (Figure 11c,d), this is an important feature to allow the microscopic inspection of the cells during the culture. The large amount of water absorbed by the scaffold in the pores and in the polymeric phase accounts for their transparency, resulting in a hydrogel structure lacking interfaces or as isotropic phase with the same refraction index as the PBS solution.

The physisorption isotherm at 77K (Figure S1) showed an adsorption branch of the isotherm typical of microporous solid and the presence of the hysteresis loop indicated the presence of mesopores. Unlike the IUPAC-classified isotherm IV, the hysteresis loop did not close at the relative pressure of 0.4, suggesting the flexible nature of the dried scaffold. The size distribution of the mesopores (defined by IUPAC in the 2–50 nm range) is shown in Figure S2, where a fraction of macropores also appears in agreement with the FESEM observations regarding the macroporous nature of the scaffolds. The BET surface area was 5.4 ± 0.2 m^2^/g and BJH adsorption cumulative pore volume of pores with diameter between 1.7 and 300 nm was 0.017 cm^3^/g.

After conditioning the scaffolds in an aqueous medium, the resulting HSs can be characterized by the [COOH]/[COO^−^] ratio, which depends on the polymer type and on the polymer/NaHCO_3_ molar ratio before thermal treatment, as well as on the pH and buffer capacity of the conditioning medium (Table 3).

POL and CBP974 polymers confer a lower density to the lyophilized scaffolds with respect to CBP980 (Table 4).

The interconnected porosity of the scaffolds is evident in SEM micrographs (Figure 9). High porosity is necessary for the migration and proliferation of cells and for the diffusion of nutrients and metabolic wastes throughout the HS. Two different methods were utilized to estimate the HS porosity, always showing values of more than 95% (Table 4), higher than the ones reported for several other hydrogel scaffolds in the literature [23,24,33].

To support the attachment of cells and to withstand the stress suffered during manipulation, the scaffolds must exhibit suitable mechanical strength and flexibility. The flexibility of the scaffolds has also been demonstrated to play a crucial role in the gene expression of cultured cells [34].

**Figure 12:**
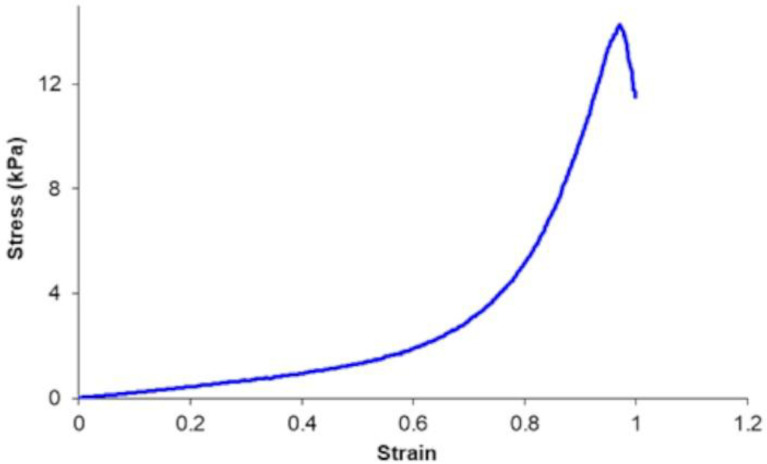
Stress–strain curve of a C0 HS.

In Figure 12, the stress–strain curve of a C0 HS conditioned in PBS is shown; the mechanical properties of scaffolds of equal composition but including different polyacrylate polymers are reported in Table 5. 

A scaffold for cell culturing should allow the free migration and interaction of the cells, besides the rapid diffusion of the cultured medium components, metabolic products, gases or vital dyes or reagents; moreover, the scaffold should also allow a very high drug diffusion, in case it has to be employed for testing the toxicological or pharmacological effects of molecules on the cells.

The diffusivity of small hydrophilic molecules has been tested at 37 °C with a home-made horizontal diffusion cell, using MH as model drug and PBS as diffusion medium. Table 6 reports the MH diffusion coefficients (D) in the three HSs studied: the measured values are reasonably consistent with values reported in the literature [35,36,37]. The diffusion coefficient of oxygen in water at 37 °C is 2.68 × 10^−5^ cm^2^/s [38], in electrospun nanofibrous polycaprolactone scaffold is 2.42 × 10^−5^ cm^2^/s and in a calcium alginate gel is 2.52 × 10^−5^ cm^2^/s [37]. The diffusion coefficient of MH in C0 was 52% of D for oxygen diffusion in water.

MH showed a slightly lower diffusion through P0 and K0 (*p* < 0.05). Also, phenol red, often used as pH indicator in cell cultures, was tested for the diffusivity, and it resulted in diffusing throughout the entire mass (approx. 850 mm^3^) of the matrix within 50 min. After 5 h, the red color was uniformly distributed throughout the PBS-conditioned HS, which proved its good permeability to this indicator. Phenol red does not cause any modifications of the chemical and physical properties of the scaffold and does not chemically react with the polyacrylic structure; in fact, it can be easily washed away with PBS.

Some coloring agents, such as MTT, MTS and NRU, used as vital dyes in cell viability tests, have been verified for compatibility and diffusion in the scaffolds. They spread into C7 (volume ~895 mm^3^) and C8 (volume ~815 mm^3^) during a 3 h incubation, evidencing the good diffusion properties of HSs and the absence of any interference between the coloring agents and the HSs.

For the same composition of scaffold the polymer CBP980, compared to POL and CBP974, provided better mechanical properties, which are desirable when handling during production steps and operation for culturing cells, while maintaining a porosity of over 95%. For this reason, and because of the better diffusion coefficient, CBP980 was selected as polymeric component of the HSs for further characterization and optimization studies and to be employed for in vitro studies.

**Figure 13:**
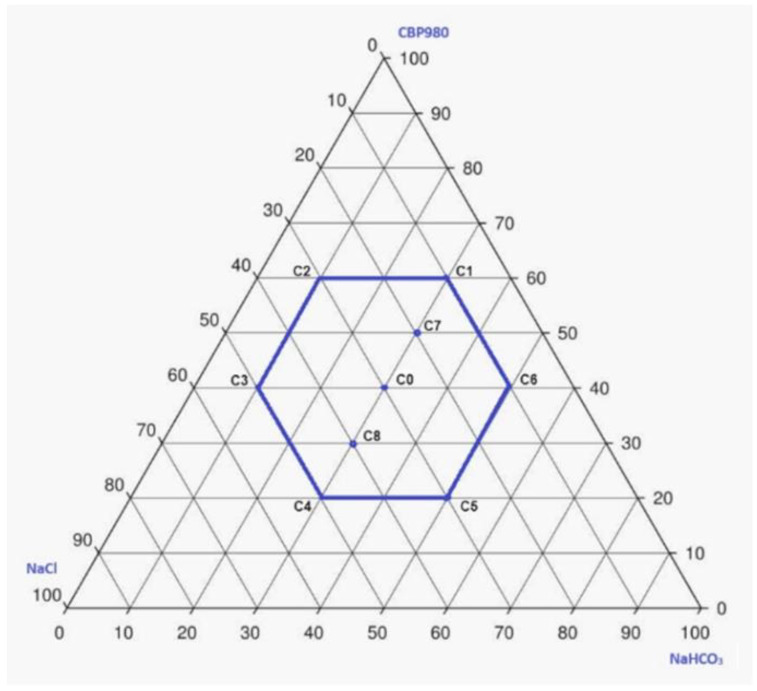
Experimental design for the HS formulations containing CBP980, NaCl and NaHCO_3._ Formulations C0-C6 were used for the mathematical modelling of the formulation properties; formulations C7 and C8 correspond to test formulations.

In order to study the effect of the different proportions of CBP980, NaCl and NaHCO_3_ on the HS properties, an experimental design for mixtures [21] was applied, considering the following relational constraints: CBP980 varied between 20 and 60%, while NaHCO_3_ and NaCl varied between 10 and 50%. This study aimed to explore the experimental domain around C0 formulation and to perform the mathematical modelling of the properties of interest, searching for optimal mixture composition. The relational constraints resulted in a smaller experimental space, contained inside the equilateral triangle domain of mixture design, with the shape of a regular hexagon. Thus, six formulations (C1–C6) were prepared and analyzed, corresponding to the vertices of the hexagon plus the central point (C0), which was replicated to estimate the experimental error. Two test points (formulations C7 and C8) were added. The experimental domain is shown in Figure 13; the initial composition of the studied formulations is given in Table 1.

Principal component analysis (PCA) was applied to the data reported in Table 7, considering eleven HSs described by five morphological parameters (weight loss after thermal treatment, diameter and thickness change after thermal treatment, diameter and thickness after leaching), two mechanical parameters (YM and YS), one chemical parameter (percentage of COO^−^) and four physical properties (SI, apparent density, compression density, porosity). Other parameters are reported in Tables S1–S3.

This multivariate statistical technique allows us to visualize in a few dimensions the complex structure of the dataset, showing the similarities among the HSs, the correlations between the different properties and the relationships between HS composition and properties.

**Figure 14:**
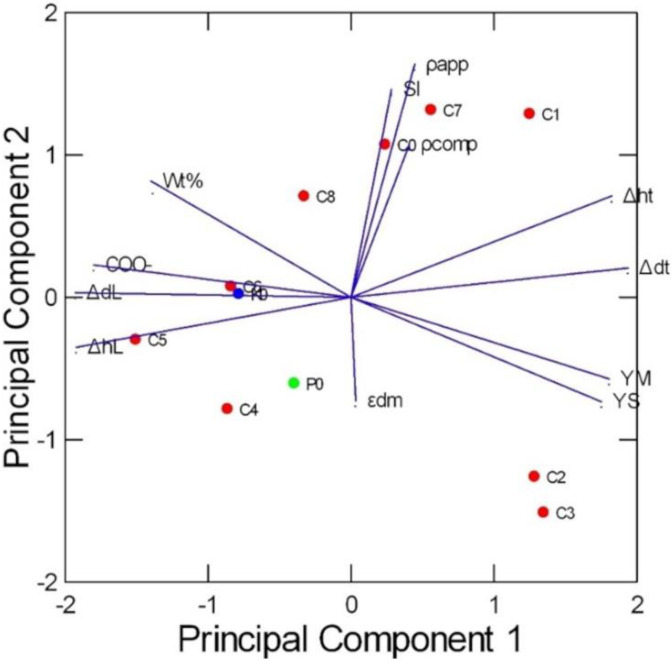
Results of the principal component analysis: biplot showing the scores of the eleven HSs and the loadings of the twelve parameters on the first two principal components (polymer used in HSs: • CBP980; • CBP974; • POL).

The biplot in Figure 14 shows the scores of the eleven HSs and the loadings of the 12 parameters. The first two principal components (PCs) explain the 73.4% of the total variance of the data (55.2% and 18.2%, respectively). Several variables (YM, YS, COO^−^ and the parameters describing the weight and size change of the HS) have high loadings on PC1, while the variables having the maximum loading on PC2 are SI and density. The diameter and thickness change after thermal treatment (Δdt and Δht) are positively correlated, as well as the diameter and thickness change after leaching (ΔdL and ΔhL): the HS size increases in both directions. A larger increase in diameter after thermal treatment corresponds to a smaller increase in diameter after leaching, i.e., Δdt and ΔdL are negatively correlated, being opposite in the plot, and are analogous for the increase in thickness, Δht and ΔhL. YM and YS are highly correlated and negatively correlated with COO^−^.

The HSs coded as P0 and K0, prepared with the same proportions of C0 but different polymers (CBP974 and POL, respectively), have different properties compared to C0, confirming the interesting characteristics of CBP980 (higher YM, YS and size variation after thermal treatment). The HSs coded as C2, C3 and C1 (high content of polymer and low content of NaHCO_3_) show the maximum values of YM and YS. Conversely, these mechanical properties have low values in C5, C8, C6 and C4 (low % of polymer and high % of NaHCO_3_). YM is an important parameter, since it is a measure of the scaffold elasticity, which can be modulated to optimize the growth of some cellular types. YS is a measure of the robustness of handling of the scaffolds. YM of the conditioned scaffolds under study ranged from 23 to 104 kPa and YS ranged from 4 to 21 kPa (Table 5). These values, which are consistent with the values reported in the literature for other hydrogel scaffolds, could offer potential in meeting the mechanical property requirements for soft tissues (YM_brain_ 0.1–1 kPa; YM_muscle_ 8–17 kPa; YM_collagenous bone_ about 100 kPa) [6].

SI and density are high in C1, C7 and C0 (at increasing polymer and decreasing NaCl content, with the same % of NaHCO_3_), while the minimum values of SI characterize C3, C4 and C2, due to the low % of NaHCO_3_.

The third and fourth PC retain the 20.4% of the variance (11.6% and 8.8%, respectively) (Figure S3). The only variable with high loading in PC3 is εdm (porosity with density method), with the minimum value for C8, which also shows low SI. In PC4, a low value of ρcomp (density for compression) characterizes C6 and C5, i.e., the HSs at the highest % of NaHCO_3_.

**Figure 15:**
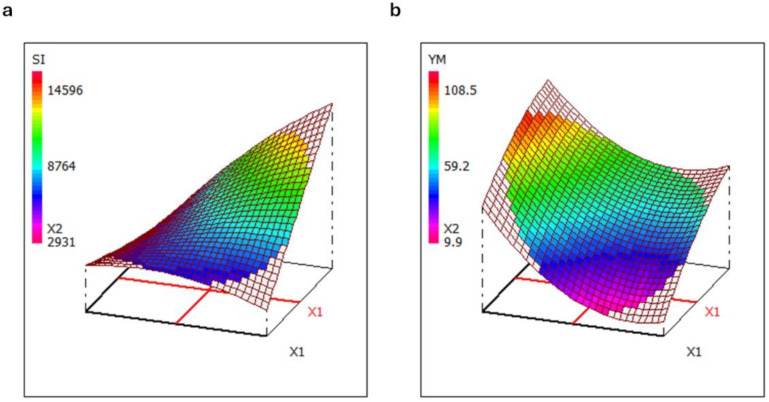
Response surface of swelling index (**a**) and of Young’s modulus (**b**).

The response surfaces obtained by mathematical modelling confirm these results. As an example, Figure 15 shows the response surfaces of SI and YM: SI increases at increasing % of CBP980 and decreasing % of NaCl. The sponge effect or swelling behavior is crucial for loading the cells in the scaffold. The NaCl content is related to microporosity, while the NaHCO_3_ content is related to mesoporosity. YM increases at the increasing % of CBP980 and the decreasing % of NaHCO_3_.

Superimposing the contour plots of the two responses, SI and YM (Figure S4), formulations showing high SI with acceptable values of YM (and also YS, with the same behavior), fall in the area of the experimental domain around C0 and C8.

**Figure 16:**
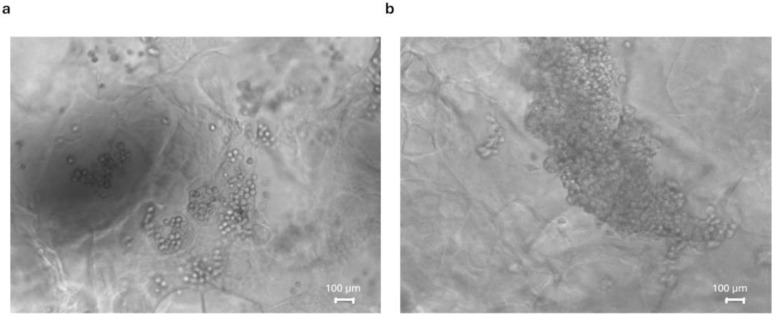
Digital images of HeLa (**a**) and HECV (**b**) cell cultures were taken after 15 days of culture in C8 HS by using a phase-contrast microscope. The figures depicted are representative of at least three similar samples (10× magnification).

A qualitative assessment of cell morphology was carried out by using a phase-contrast microscope at 10× magnification every 48 h. The scaffold transparency allowed us to confirm the healthiness of the cultures: no evident alterations of cell morphology or necrotic figures were observed through contrast phase microscopy throughout the 20 days of culture. The digital images in Figure 16 show 15 days of culture as representatives of cell capability to aggregate into clusters and develop complex structures.

**Figure 17:**
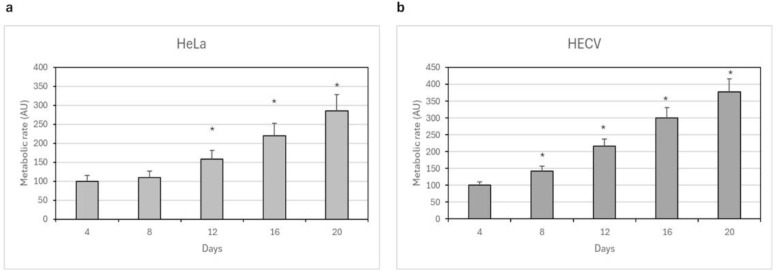
Healthy state of HeLa (**a**) and HECV cells (**b**) during culture in C8 HS (diameter × height 6 × 5 mm) estimated by the Alamar Blue test. Data are expressed as the means of three separate experiments ± standard deviation, run in triplicate. Data are expressed as arbitrary units (AUs), referring to a percentage relative to the respective value obtained at day 4. * *p* < 0.01 versus the respective AU at day 4 (ANOVA followed by Dunnett’s tests).

The twenty day-long viability of human HeLa and endothelial HECV cells cultured in C8 HS was assessed by Alamar Blue. The healthy state index (Figure 17) evidenced an increase in metabolic state up to 20 days of culture, confirming that the scaffold did not exert toxic effects.

**Figure 18:**
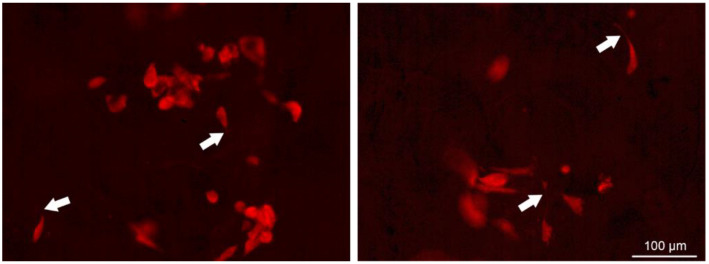
Sections from the C8 scaffold including the HECV cell line, agarose-embedded, and PI-stained. Both the cytoplasm and the nucleus show red, as PI binds to both DNA and RNA. The scaffold is hardly visible under the used wavelengths. Cells exhibit different shapes and processes, as indicated by the arrows.

**Figure 19:**
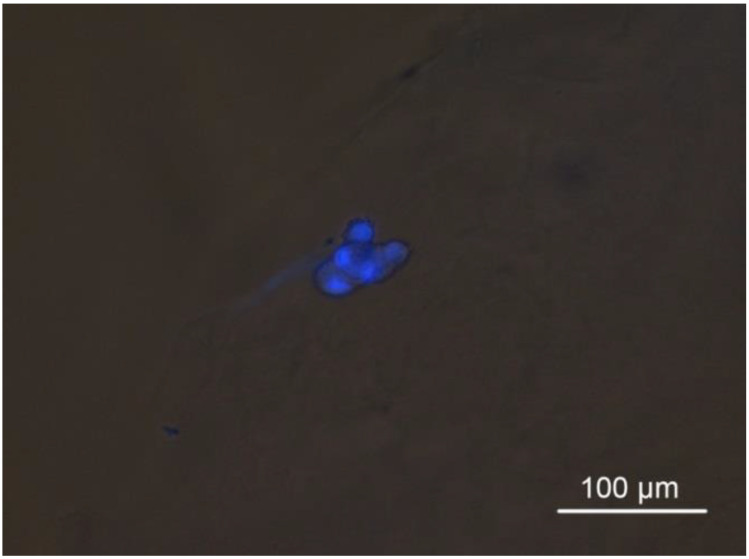
Epifluorescence microscopy combined with transmitted light and differential interference contrast filters. Sections from the C7 scaffold including the HECV cell line, agarose-embedded, DAPI-stained. The nuclei show fluorescence as expected, and the scaffold is invisible under the used wavelengths.

**Figure 20:**
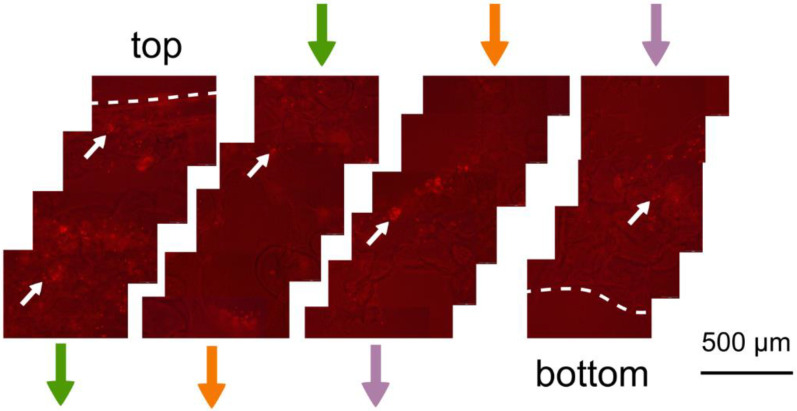
Epifluorescence microscopy sections from the C7 scaffold, including the HeLa cell line, agarose-embedded, and PI-stained; the sequence of the sections ranges from the seeding surface (top) and continues (following the “path” indicated by the arrows with the same color) down to the bottom of the scaffold. Cells (white arrows) are evenly distributed. The dashed lines indicate the upper and lower edges of the scaffold section. A red background is visible, though it does not hamper cell identification and observation. This is due to the longer exposure time required at low magnification during micrograph acquisition.

All cells cultured in HSs colonized the scaffolds from the seeding surface to the bottom, as shown by the fluorescent imaging of fixed cells in HSs embedded in agarose. The acquired micrographs allow us to observe cell shape and distribution and, moreover, confirm the compatibility of the HS with fluorescence microscopy, which is an essential tool in cell biology (Figure 18, Figure 19 and Figure 20).

**Figure 21:**
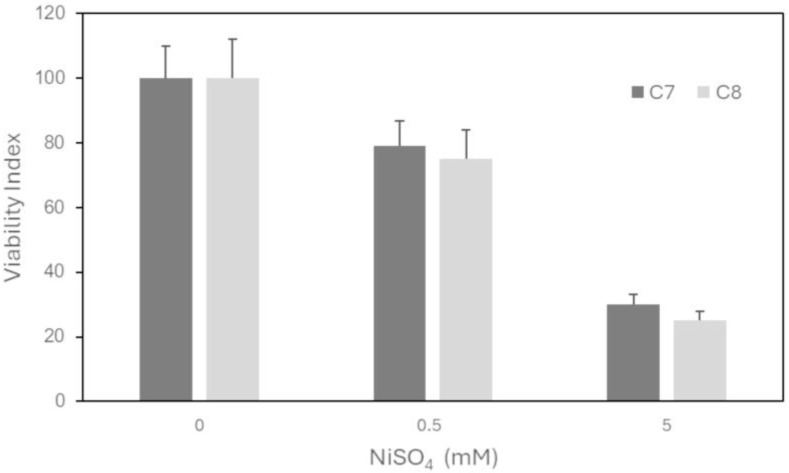
Effect on viability of HeLa cells cultured in C7 and C8 scaffolds and exposed to NiSO_4_ for 3 h, as the positive control of irritation; the viability indexes are derived from the means of two genuine replicates performed in duplicate ± standard deviation.

To verify whether the HS can represent a useful tool to perform a prediction cytotoxicity test, HeLa cells embedded in C7 and C8 HSs were exposed to 0.5 and 5 mM aqueous NiSO_4_ solutions as a positive irritation agent. The cells showed a significant decrease in MTS viability index in a dose-dependent manner, without relevant difference between the two types of scaffolds. The obtained results suggested the suitability of the developed HSs for irritation test with NiSO_4_ (Figure 21).

## 4. Conclusions

Three-dimensional cell culture technologies significantly paved the way to the overcoming of the limitations of two-dimensional methods in cell biology and biochemistry fields, as well as in pharmaceutical assays. In this context, a novel 3D hydrogel sponge scaffold, composed of a crosslinked polyacrylic acid forming a porous matrix, was developed and characterized. The scaffold was obtained via an innovative procedure involving thermal treatment followed by a salt-leaching step on a matrix containing polymer along with a gas-forming agent. Based on experimental designs for mixtures, a series of formulations were prepared with the aim of studying the effects of the different components (NaHCO_3_, polyacrylic acid and NaCl) on the scaffold properties. The best compositions were selected by considering the scaffold mechanical properties, density, swelling behavior, carboxylic acid salification and morphological changes. Other characteristics such as physical appearance, surface morphology, transparency, diffusion properties, porosity, biocompatibility and cytocompatibility were also evaluated. 

The obtained sponge scaffolds exhibited high porosity, optical transparency, good mechanical resistance and, noteworthy, the ability to maintain different cell lines in culture for at least 20 days.

For further evaluation, the scaffolds might be coated or treated with specific growth factors, conferring enhanced biocompatibility on certain specific cellular types. Considering the presence of carboxylic acidic functions on the scaffold, it is conceivable that ionic or covalent bonds might be exploited to stabilize some of these factors, like the natural components of the extracellular matrix. Following the same principle, the scaffold might be converted into a suitable matrix for bone cell growth by coating with osteogenic proteins. Their excellent mechanical properties also suggest a possible use in bioreactors. 

In conclusion, the developed hydrogel sponge scaffolds represent an important alternative tool to obtain a more predictive cellular response during drug evaluation in the context of preclinical drug development studies, allowing a reduction in animal testing. Moreover, if suitably adapted, these scaffolds could represent a support for the establishment of alternative methods for the toxicological study of chemical compounds used in different products, like biocides and cosmetics.

## Figures and Tables

**Table 1:** List of different HSs prepared, including the code and the initial composition of each powder mixture.

HS Code	CBP980 (%)	CBP974 (%)	POL (%)	NaHCO_3_ (%)	NaCl (%)
C0	40			30	30
C1	60			30	10
C2	60			10	30
C3	40			10	50
C4	20			30	50
C5	20			50	30
C6	40			10	50
C7	50			30	20
C8	30			30	40
K0		40		30	30
P0			40	30	30

**Table 2:** Characteristics of three 13 × 1.3 mm cylindrical compact unit of scaffolds with the same compositions but using different polymers for scaffold preparation (CBP980 coded as C; CBP974, coded as K; POL coded as P), submitted to the same thermal treatment; weight loss (W_t_%), diameter and thickness variation after thermal treatment (Δdt and Δht, respectively), diameter and thickness variation after leaching (ΔdL and ΔhL, respectively), reported as mean value ± standard deviation (n = 6).

HS Code	W_t_%	Δdt (%)	Δht (%)	ΔdL (%)	ΔhL (%)
C0	12.3 ± 0.4	50.6 ± 8.7	130.3 ± 27.3	215.4 ± 17.0	133.4 ± 29.7
K0	10.7 ± 0.9	17.2 ± 4.5	49.8 ± 10.9	346.7 ± 20.4	251.6 ± 31.7
P0	10.4 ± 1.0	26.2 ± 2.6	74.2 ± 15.5	267.1 ± 11.0	179.0 ± 2 7.3

**Table 3:** Value of COO^−^ (%) of three different HSs.

HS Code	COO^−^ (%)
C0	90.0
K0	83.4
P0	86.1

**Table 4:** Apparent density (ρapp), compression density (ρcomp) measured on lyophilized HSs, and porosity calculated by density method (εdm) and measured by liquid displacement (εld). Data are reported as mean value ± standard deviation (n = 6).

HS Code	ρapp (mg/cm^3^)	ρcomp (mg/cm^3^)	εdm (%)	εld (%)
C0	20.9 ± 1.0	1702.3 ± 21.6	97.90 ± 0.08	97.63 ± 0.35
K0	15.8 ± 0.3	1424.1 ± 53.9	99.29 ± 0.02	98.33 ± 0.15
P0	11.2 ± 0.5	1568.9 ± 31.6	98.91 ± 0.04	96.21 ± 0.95

**Table 5:** Mechanical properties of 20 mm diameter cylindrical and PBS conditioned HSs, completely immersed in PBS. Data are reported as mean value ± standard deviation (n = 6).

HS Code	PBS Conditioned Weight (mg)	PBS Conditioned Thickness (mm)	Young’s Modulus (kPa)	Yield Strength (kPa)
C0	1780.2 ± 148.6	4.83 ± 0.12	52.4 ± 1.9	11.7 ± 1.3
K0	1587.7 ± 22.5	5.15 ± 0.10	28.3 ± 1.2	6.3 ± 0.2
P0	1599.3 ± 51.4	5.37 ± 0.10	38.7 ± 2.1	7.9 ± 0.3

**Table 6:** MH diffusion coefficients in HSs in PBS at 37 °C (mean values ± standard deviation, n = 6).

HS Code	D (cm^2^/s)
C0	1.37 × 10^−5^ ± 3.4 × 10^−7^
K0	1.25 × 10^−5^ ± 1.0 × 10^−7^
P0	1.16 × 10^−5^ ± 9.3 × 10^−7^

**Table 7:** Properties of the different HSs used for principal component analysis: percentage weight loss after thermal treatment (W_t_%), diameter change (Δdt) and thickness change (Δht) after thermal treatment, diameter change (ΔdL) and thickness change (ΔdL) after leaching, swelling index (SI), Young’s modulus (YM), yield strength (YS), apparent density (ρapp), compression density (comp), porosity by density method (εdm), and % of carboxylates (COO^−^). Except for COO^−^, data are reported as average value ± standard deviation (n = 6).

HS Code	W_t_ %	Δdt %	Δht %	ΔdL %	ΔhL %	SI %	YM kPa	YS kPa	ρapp mg/cm^3^	ρcomp mg/cm^3^	εdm %	COO^−^ %
C0	12.3 ± 0.4	50.6 ± 8.7	130.3 ± 27.3	215.4 ± 17.0	133.4 ± 29.7	7937.6 ± 384.1	52.4 ± 1.9	11.7 ± 1.3	20.9 ± 1.0	1702.3 ± 21.6	97.90 ± 0.08	90.03
C1	12.8 ± 0.6	102.1 ± 47.4	240.7 ± 31.1	172.6 ± 65.1	69.3 ± 9.8	12394.1 ± 277.0	68 ± 2.0	12.4 ± 0.7	17.4 ± 0.7	1598.6 ± 27.1	98.78 ± 0.07	50.58
C2	5.6 ± 0.3	88.8 ± 5.2	156.5 ± 17.2	129.1 ± 6.7	99.1 ± 12.0	5882.5 ± 343.8	73.4 ± 6.3	20.0 ± 1.7	13.5 ± 0.5	1384.9 ± 24.9	99.03 ± 0.04	50.82
C3	5.4 ± 0.1	75.8 ± 3.2	149.6 ± 14.5	141.6 ± 5.6	90.0 ± 8.8	4966.5 ± 169.8	104.1 ± 5.0	20.9 ± 0.7	14.8 ± 0.3	1238.7 ± 25.6	98.79 ± 0.03	57.44
C4	12.3 ± 0.3	5.1 ± 1.8	39.7 ± 11.8	346.8 ± 24.6	235.5 ± 28.6	5688.2 ± 529.6	39.0 ± 2.3	7.4 ± 0.7	12.7 ± 1.2	1565.6 ± 18.0	98.68 ± 0.06	88.87
C5	20.0 ± 0.1	1.7 ± 0.7	20.5 ± 5.9	366.5 ± 11.1	320.7 ± 39.3	6359.2 ± 524.2	23.3 ± 5.4	4.5 ± 1.2	15.2 ± 1.0	1190.3 ± 22.3	98.64 ± 0.06	94.59
C6	21.5 ± 0.9	20.4 ± 4.9	71.1 ± 15.1	291.0 ± 5.1	224.0 ± 24.8	8689.5 ± 824.2	40.2 ± 3.9	9.7 ± 0.9	15.3 ± 0.6	1126.9 ± 12.3	98.77 ± 0.02	99.31
C7	12.8 ± 0.8	58.9 ±15.3	177.4 ± 34.0	213.6 ± 24.6	101.7 ± 24.2	9901.9 ± 454.3	53.3 ± 5.9	10.3 ± 0.6	20.3 ± 0.9	1573.3 ± 39.8	98.90 ± 0.04	65.45
C8	12.9 ± 0.3	25.6 ± 7.5	83.4 ± 17.9	246.2 ± 40.0	175.3 ± 40.1	5821.6 ± 992.3	38.8 ± 5.2	9.8 ± 1.0	17.6 ± 0.6	1673.8 ± 5.2	99.17 ± 0.10	93.64
K0	10.7 ± 0.9	17.2 ± 4.5	49.8 ± 10.9	346.7 ± 20.4	251.6 ± 31.7	9878.8 ± 359.1	28.3 ± 1.2	6.3 ± 0.2	15.8 ± 0.3	1424.1 ± 53.9	99.29 ± 0.02	83.45
P0	10.4 ± 1.0	26.2 ± 2.6	74.2 ± 15.5	267.1 ± 11.0	179.0 ± 27.3	6900.5 ± 412.8	38.7 ± 2.1	7.9 ± 0.3	11.2 ± 0.5	1568.9 ± 31.6	98.91 ± 0.04	86.05

## Data Availability

Data are contained within the article or the Supplementary Materials.

## Supplementary Materials

The following are available online at https://www.mdpi.com/article/10.3390/pharmaceutics16101341/s1. Figure S1: Graph representing an isothermal plot for lyophilized C0 HS; Figure S2: Graph representing pore size distribution by BJH method for lyophilized C0 HS; Figure S3: Results of principal component analysis; Figure S4: Contour plots of swelling index and of Young’s modulus; Table S1: Value of COOH and COO^−^ (meq/mg) and COO^−^ percentage of the different HSs; Table S2: Porosity by the density method and liquid displacement of the different HSs; Table S3: Morphological and mechanical properties of the different HSs.
