# Supplementary material for: A Novel Hydrogel Sponge for Three-Dimensional Cell Culture
Source: Pharmaceutics. 2024 Oct 19;16(10):1341. doi: 10.3390/pharmaceutics16101341 (PMC11511160; doi:10.3390/pharmaceutics16101341)
Supplement: Supplementary file 1 [file pharmaceutics-16-01341-s001.zip › pharmaceutics-3244598-supplementary.pdf]

pharmaceutics

Article

Supplementary Materials: A Novel Hydrogel Sponge for Three-Dimensional Cell Culture

Sara Baldassari, Mengying Yan, Giorgia Ailuno, Guendalina Zuccari, Anna Maria Bassi, Stefania Vernazza, Sara Tirendi, Sara Ferrando, Antonio Comite, Giuliana Drava and Gabriele Caviglioli

S1. Methods

S1.1 HS Lyophilization Procedure

The swollen scaffolds, conditioned in water or PBS, were lyophilized in a Freeze Dry System (Labconco, Kansas City, USA), at a pressure lower than 20 mbar, primary drying at –30 °C for 48 h, secondary drying at 25 °C for at least 1 h. After freeze-drying, the scaffolds were maintained in a desiccator.

S1.2 Experimental Design and Response Surface Methodology

Lower and upper limits were set for each component: CBP980 was varied between 20 and 60%, NaCl and $NaHCO_3$ between 10 and 50%. These constraints resulted in an experimental space having the shape of a regular hexagon inside the ternary diagram, as shown in Figure 13. This experimental domain was explored by preparing 6 formulations (C1–C6, Table 1) corresponding to the vertices of the hexagon plus the central point (C0), which was replicated to provide an estimate of the experimental error. The formulations C7 and C8 were used as test points. The mathematical function relating the formulations composition with each measured response is a second-order model whose coefficients were estimated by least squares multilinear regression and allowed to obtain response surfaces for optimization purpose.

S1.3 Mechanical Properties

A compression test was performed on 20 mm-diameter cylindrical HSs, conditioned and sterilized in PBS, using a 20 N load cell and a cylindrical (r = 5.82 mm) test probe. The tested HSs were completely immersed in PBS with 1 mm of liquid over the scaffold surface; the probe was lifted to contact with the flat surface of the HS and the elongation was zeroed, then the HSs were compressed (compression rate 1 mm/min) up to break. Stress-strain curves were recorded, with stress calculated as the force divided by the probe area in contact with the scaffold surface, and the strain as the absolute deformation divided by the scaffold initial thickness.

S1.4 Apparent and Compression Density Study

For apparent density (ϱapp), water-conditioned scaffolds, after sterilization, were cut into cylindrical shape with 15–20 mm diameter. After lyophilization, volume and weight were measured, and the apparent density calculated. For compression density (ϱcomp), 300 mg lyophilized HSs were accurately weighed in a 13 mm cylindrical die and compressed at 8 tons for 15 min in oleo-dynamic press, evacuating air by pump, volume and weight were measured and compression density calculated.

S1.5 HS Porosity

The HSs are very light, thus evaluating the volume occupied by acetonitrile within the scaffold with a pycnometer, as the liquid displacement method normally requires, is inapplicable: for this reason, the HSs were first placed in acetonitrile at 20 ± 0.5 °C and air

bubbles were evacuated under vacuum, until the complete sinking of scaffolds in the liquid. The HSs were then collected, patted with filter paper to remove excess liquid, and weighed. The recovered HSs were dried at 80 °C in forced-air oven up to constant weight, generally for approx. 40 min.

S1.6 Molecular Diffusion

PBS-swollen scaffolds (15 mm diameter and thickness ranging from 3 to 7 mm) were used for the diffusion studies. The scaffolds were placed between the two compartments, fitted into a PTFE ring of 14 mm internal diameter, measuring the resulting increased thickness of the HS. 21.5 mL of PBS solution preheated at 37 °C were placed in the receptor compartment, while the donor compartment was filled with 21.5 mL of a 0.5 mg/mL MH solution in PBS, preheated at 37 °C. Aliquots (500.0 µL) were withdrawn from both compartments at 45 min and 3, 6, 21, 24, 27 and 30 h, and MH concentration was assayed by UV absorbance (HP8452 spectrophotometer, Perkin-Elmer, Waltham, USA) at λ = 233 nm, against a 5.0 µg/mL MH standard solution. At any sampling time t, MH concentrations in the two chambers were measured to calculate the diffusion coefficient D.

Phenol red diffusivity was also tested: one drop of 1 mg/L phenol red solution was dripped on the surface of a cylindrical HSs (approx. volume 615 mm^3) conditioned with PBS; the diffusion of the red color within the matrix was evaluated visually.

S1.7 Cell Viability Tests

PBS-conditioned HSs were maintained in DMEM medium until the assays were performed. Each HS was placed into a 6-multiwell plate containing 2 mL of NRU, MTS and MTT reagents in duplicate, to evidence if any interaction between dyes and HS may occur. For the NRU assay, cell cultures were exposed to culture medium containing 50 µg/mL Neutral Red dye (without serum and phenol red). After a 3 h incubation at 37 °C, the cells were washed twice with PBS and bleached. The HS cytotoxic effects and the cell proliferation were assessed by MTS assay (CellTiter 96® Aqueous One Solution Cell Proliferation Assay, Promega, Madison, USA), measuring the optical density (OD) of the solutions of the dissolved formazan crystals and the released Neutral Red dye (for the MTS and NRU tests, respectively) at 570 nm with a Uniskan II Microplate reader (Labsystems, Vantaa, Finland). Cell healthiness was evaluated by Alamar Blue® assay. To assess the optimal time to measure fluorescence after addition of Alamar Blue® in wells, preliminary measurements were performed at 1, 2 and 3 h by withdrawing 10 µL of experimental medium. The obtained results suggested that a 3-h incubation time is optimal. For cell culture studies, HSs were reconstituted into single wells of a 24 well plates and conditioned in DMEM. The different cells studied were seeded into the conditioned HSs (100–200 × 10^3 cellular density) in 500 µL DMEM medium supplemented with 10% FBS, 2 mM glutamine, and were cultured in a humidified atmosphere containing 5% CO_2 at 37 °C. At each check point time, using sterile plastic forceps with flattened tips the scaffolds were transferred into another 24-well plate, gently washed with PBS and then treated with 1 mL fresh medium added with 10% Alamar Blue® reagent. Fluorescence was measured at the end of the incubation time ($\lambda_{excitation}$ = 530 nm, $\lambda_{emission}$ = 590 nm). As positive controls, wells containing culture medium with 10% v/v Alamar Blue® and vitamin C (ascorbic acid 0.75 mg in 5 µL/well, which causes rapid full reduction of Alamar Blue® (Invitrogen, Thermo Fisher Scientific Inc., Waltham, MA, USA)), in the absence of cells and hydrogel, were used; as negative controls, wells with culture medium containing 10% v/v Alamar Blue®, but not vitamin C and without cells, were used.

S1.8 Fluorescent Imaging of Fixed Cells in HS Embedded in Agarose

Utilizing a vibratome (MA752 Motorized Advance Vibroslice Microtome, Campden Instruments, Loughborough, UK), 40 µm-thick sections of agarose-embedded HSs containing cultured cells were prepared. These sections were mounted onto histological slides

and subsequently stained with two commonly used fluorescent dyes that target nucleic acids.

Propidium iodide (PI) binds both DNA and RNA in fixed cells; when bound, its excitation peak is at 535 nm (green) and its emission peak is at 617 nm (red). The PI stock solution (1 mg/mL in water) was aliquoted and stored at –20 °C. The PI working solution was prepared as follows: 2 µL of PI were added to 2 mL of PBS 8‰ NaCl and pH 7.4. Sections were exposed to 1 mL of working solution for 40 min at room temperature in the dark, after which the slides were gently washed several times with PBS.

Alternatively, sections were stained with 4′,6-diamidino-2-phenylin-dole (DAPI), which binds DNA by intercalation and, when bound, it emits blue fluorescence (465 nm) when excited by proper wavelength (UV, 350 nm). The DAPI stock solution (1 mg/mL) was prepared as follows: 10 mg of DAPI (Molecular Probes, Cat# D-1306) were added to 1 mL of dimethylsulfoxide, then to 9 mL of ethanol absolute, and mixed to complete dissolution, aliquoted, and stored at –20 °C. The DAPI working solution was prepared by diluting 1:1000 in PBS and stored at 4 °C in a dark bottle. Sections were incubated for 10 min at room temperature in the dark with the working solution. As DAPI is not fluorescent when unbound, no washing step was required after staining.

After either PI or DAPI treatment, slides were mounted in PBS with cover slip; glycerol, which is generally used in the mounting media, was not employed to avoid interactions with the hydrogel. Then the slides were observed through a Leica DMRB light and epifluorescence microscope equipped with differential interference contrast filters, a Mercury Short Arc Ushio USH-102/D 100 W lamp (Ushio Europe, Oude Meer, The Netherlands) and A (blue fluorescence) and N2.1 (red fluorescence, excitation ~530–560 nm, emission > 590 nm) filter cubes. Micrographs were acquired by a Leica CCD camera DFC420C (Leica, Wetzlar, Germany) and Leica Application Suite (LAS) software (version 5.2.2).

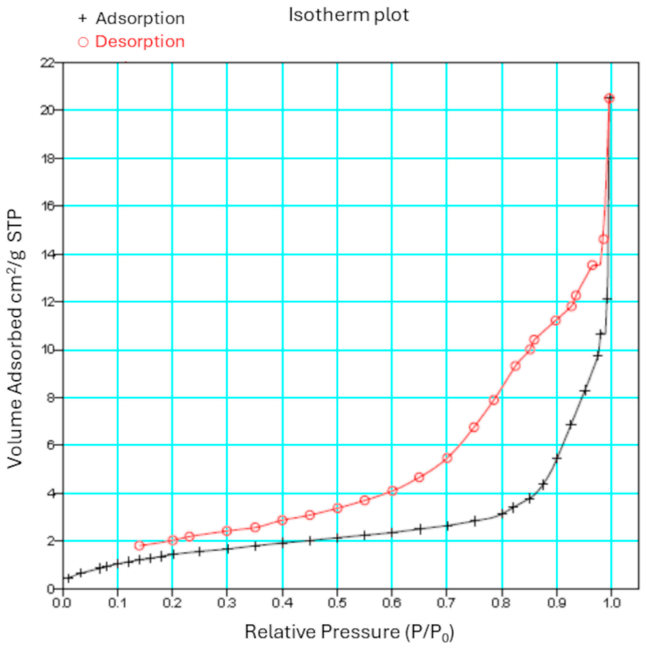

Figure S1. Graph representing an isothermal plot for lyophilized C0 HS.

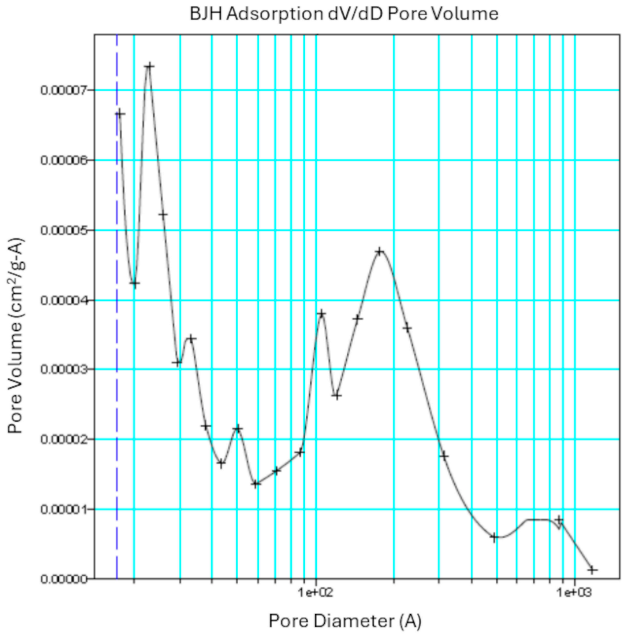

Figure S2. Graph representing pore size distribution by BJH method for lyophilized C0 HS: dV/dD pore volume (+) against the pore diameter.

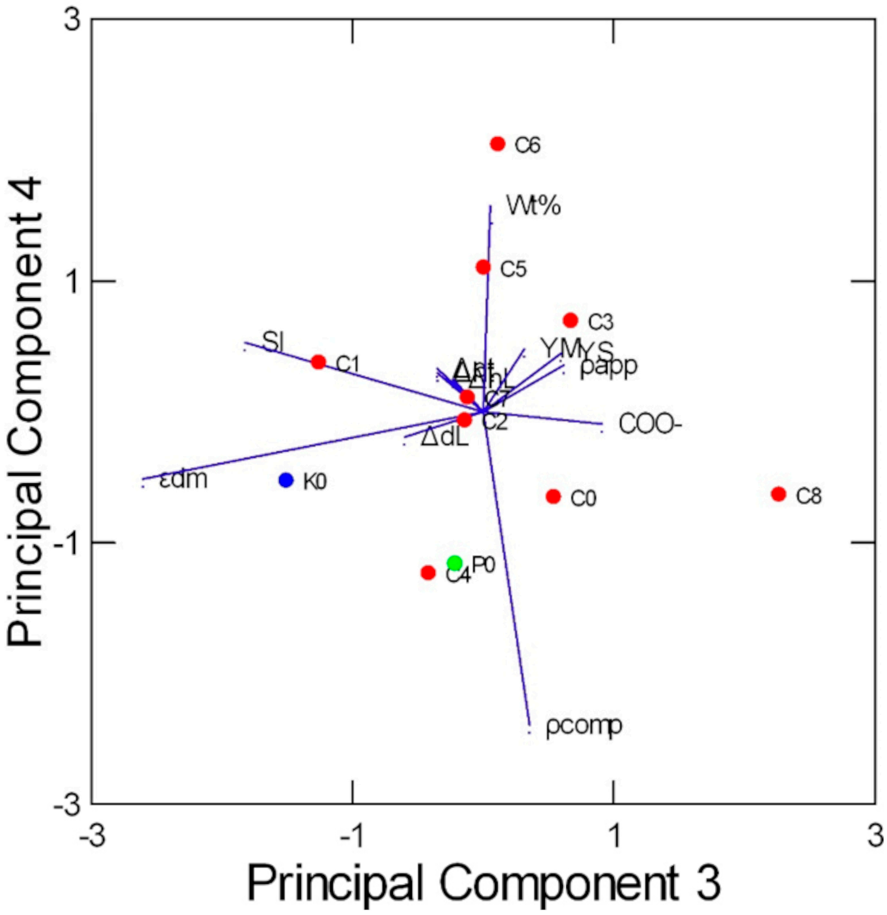

Figure S3. Results of Principal Component Analysis: biplot showing the scores of the eleven HSs and the loadings of the 12 parameters on the third and fourth Principal Component (polymer used in HS: • CBP980; • CBP974; • POL).

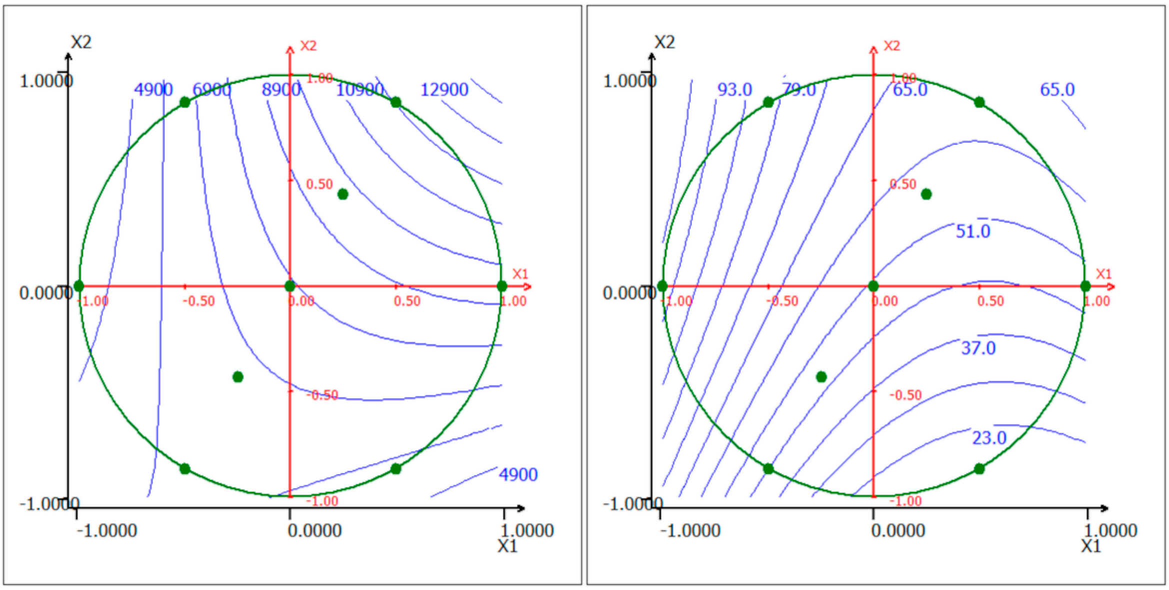

Figure S4. Contour plots of Swelling Index (left) and of Young's modulus (right). By superimposing the contour plots, the HSs having high SI and acceptable YM are C0 and C7. The green points indicate the HS formulations; the blue lines are the iso-response curves; the red lines indicate the axes of the plane containing the experimental domain shown in Figure 13.

Table S1. Value of COOH and COO^- (meq/mg) and COO^- percentage of the different HSs.

HS code	Polymer/Na-HCO_3	COOH meq/mg	COO^- meq/mg	Total meq/mg	COO^- %
C0	1.3	0.0000982	0.008868	0.009851	90.03
C1	2	0.0050254	0.005143	0.010169	50.58
C2	6	0.0047358	0.004895	0.009631	50.82
C3	4	0.0043133	0.005822	0.010136	57.44
C4	0.6	0.0010304	0.008230	0.009260	88.87
C5	0.4	0.0005104	0.008917	0.009428	94.59
C6	0.8	0.0000513	0.007935	0.007990	99.31
C7	1.7	0.0033421	0.006332	0.009674	65.45
C8	1	0.0006230	0.009180	0.009803	93.64
K0	1.3	0.0014779	0.007452	0.008930	83.45
P0	1.3	0.0011951	0.007370	0.008565	86.05

Table S2. Porosity by density method and by liquid displacement of the different HSs: data are reported as average value ± standard deviation (n = 6).

HS code	ϱapp mg/cm^3	ϱcomp mg/cm^3	εdm %	εld %
C0	20.9 ± 1.0	1702.3 ± 21.6	97.90 ± 0.08	97.63 ± 0.35
C1	17.4 ± 0.7	1598.6 ± 27.1	98.78 ± 0.07	97.87 ± 0.29
C2	13.5 ± 0.5	1384.9 ± 24.9	99.03 ± 0.04	98.56 ± 0.10
C3	14.8 ± 0.3	1238.7 ± 25.6	98.79 ± 0.03	98.33 ± 0.09
C4	12.7 ± 1.2	1565.6 ± 18.0	98.68 ± 0.06	97.99 ± 0.18
C5	15.2 ± 1.0	1190.3 ± 22.3	98.64 ± 0.06	97.89 ± 0.21
C6	15.3 ± 0.6	1126.9 ± 12.3	98.77 ± 0.02	97.23 ± 0.53
C7	20.3 ± 0.9	1573.3 ± 39.8	98.90 ± 0.04	97.94 ± 0.14
C8	17.6 ± 0.6	1673.8 ± 5.2	99.17 ± 0.10	98.33 ± 0.10
K0	15.8 ± 0.3	1424.1 ± 53.9	99.29 ± 0.02	98.33 ± 0.15

P0	11.2 ± 0.5	1568.9 ± 31.6	98.91 ± 0.04	96.21 ± 0.95

Table S3. Morphological and mechanical properties of the different HSs: data are reported as average value (n = 6) ± standard deviation.

HS code	PBS conditioned weight (mg)	PBS conditioned thickness (mm)	Young's modulus (kPa)	Yield strength (kPa)
C0	1780.2 ± 148.6	4.83 ± 0.12	52.4 ± 1.9	11.7 ± 1.3
C1	2152.7 ± 113.7	6.77 ± 0.17	68 ± 2.0	12.4 ± 0.7
C2	2157.3 ± 70.56	6.33 ± 0.06	73.4 ± 6.3	20.0 ± 1.7
C3	1685.0 ± 77.06	5.30 ± 0.13	104.1 ± 5.0	20.9 ± 0.7
C4	1155.3 ± 116.2	3.43 ± 0.38	39.0 ± 2.3	7.4 ± 0.7
C5	958.3 ± 43.2	3.27 ± 0.29	23.3 ± 5.4	4.5 ± 1.2
C6	1763.9 ± 123.6	5.64 ± 0.06	40.2 ± 3.9	9.7 ± 0.9
C7	1801.3 ± 79.4	6.18 ± 0.20	53.3 ± 5.9	10.3 ± 0.6
C8	1461.0 ± 114.5	4.91 ± 0.31	38.8 ± 5.2	9.8 ± 1.0
K0	1587.7 ± 22.5	5.15 ± 0.10	28.3 ± 1.2	6.3 ± 0.2
P0	1599.3 ± 51.4	5.37 ±0.10	38.7 ± 2.1	7.9 ± 0.3
